# Ebola’s hidden front: Neurological involvement and pathogenesis

**DOI:** 10.1371/journal.ppat.1013725

**Published:** 2025-11-21

**Authors:** Blake Bernauer, Jack Siemering, Chen Sabrina Tan

**Affiliations:** 1 Department of Medicine, University of Iowa, Iowa City, Iowa, United States of America; 2 Program in Immunology, University of Iowa, Iowa City, Iowa, United States of America; University of Iowa, UNITED STATES OF AMERICA

## Introduction

Survivors of Ebola virus disease (EVD), one of the most severe viral infections known to humans, are often burdened with late-onset neurological complications from weeks to months after recovering. Caused by a negative-strand RNA virus of the *Filoviridae* family, EVD is notorious for its acute presentation of fever, fatigue, vomiting, and hemorrhagic complications [1,2]. Since its discovery in 1976, Ebola virus (EBOV) has caused multiple outbreaks with average case fatality rate of ~45%, including the devastating 2013–2016 Western African epidemic, which claimed over 11,000 lives and left tens of thousands of survivors at risk for chronic health issues [3].

Beyond the acute phase, increasing reports have now highlighted a spectrum of long-term neurological complications in survivors. These include meningoencephalitis, migraines, stroke, peripheral neuropathy, and neurocognitive dysfunction [4]. These chronic manifestations and EVD sequelae also include more general symptoms such as headaches, depression, arthralgia, and visual disturbances [5,6].

Despite growing recognition of these chronic neurological complications, the biological underpinnings of EVD sequelae remain poorly understood. There is a striking disconnect between the breadth of reported symptoms and our limited knowledge of EBOV neuropathogenesis in the central nervous system (CNS). Key questions persist: How and when does EBOV gain access to the CNS? What mechanisms might allow it to persist within CNS tissue? And what mechanisms drive long-term neurological symptoms, such as direct viral effects, immune-mediated damage, or other sequelae of systemic infection?

Importantly, distinguishing between persistent viral replication, reactivation of dormant EBOV, and immune-driven injury remains a challenge. Some EVD sequelae may stem from sustained neuroinflammation even in the absence of active virus, resembling post-viral syndromes described for other neurotropic infections. Persistent infection within the CNS, an immune-privileged environment, could have particularly severe consequences for cognition, behavior, and long-term function. These biological and clinical uncertainties also carry major public-health implications, as viral persistence in survivors has been linked to stigma, sexual transmission, and rare instances of outbreak re-ignition.

This review synthesizes the current understanding of EBOV neuroinvasion and persistence, explores their implications for post-acute neurological outcomes, and highlights key gaps in knowledge that must be addressed to improve survivor care and inform future research. Key research gaps and potential strategies to address them are summarized in Table 1.

**Table 1:** Major knowledge gaps and future research priorities in Ebola virus neuropathogenesis.

Knowledge gap	Current limitation	Future research / approach
**Mechanisms of CNS entry**	Limited patient tissue; uncertainty between BBB disruption and immune cell “Trojan horse” routes	Multiplex viral and immune-cell imaging in NHPs; develop BBB-on-chip and endothelial organoid models
**Mechanisms of viral persistence**	Unclear whether CNS viral persistence reflects latency or low-level replication	Longitudinal samples and single-cell analyses from survivor samples; in vitro infection of neural and glial cultures
**Model feasibility**	Modified immunocompromised murine model may not fully reflect human viral pathogenesis. BSL-4 containment restricts long-term or behavioral studies in NHPs	Employ NAMs such as organoids, organ-on-chip, targeted modifications of murine models, and pseudotyped viral systems for entry studies or recombinant surrogate models such as rVSV-EBOV-GP systems to complement in vivo work
**Neurocognitive sequelae in survivors**	Sparse standardized follow-up and neuroimaging in endemic regions	Integrate cognitive testing and imaging within survivor cohorts (e.g., PREVAIL III, PostEbogui)
**Public-health implications**	Stigma, relapse, and inadequate surveillance impede survivor reintegration	Establish coordinated survivor support, monitoring, and outbreak-prevention programs

Legend: BBB, blood–brain barrier; CNS, central nervous system; NAMs, new approach methodologies; NHPs, nonhuman primates; rVSV-EBOV-GP, recombinant vesicular stomatitis virus expressing Ebola glycoprotein.

## Q1: How does Ebola virus reach the brain?

A critical step in understanding the neurological impact of EVD is elucidating how the virus reaches and infects the CNS. Current evidence suggests that EBOV gains access to the brain through a combination of direct infection, immune cell trafficking, and disruption of the blood–brain barrier (BBB) [7,8].

EBOV typically attaches to host cells via factors such as T-cell immunoglobulin and mucin domain-1, while viral entry requires the endosomal receptor Niemann-Pick C1, which facilitates fusion of the viral and endosomal membranes [9,10]. Within the CNS, endothelial cells of the BBB and choroid plexus express these receptors and could serve as potential points of viral entry [11]. Additionally, EBOV glycoprotein has been shown to disrupt β1 integrins on endothelial cells, potentially compromising BBB integrity and facilitating paracellular or transcellular passage of viral material to the brain [12].

This disruption may be exacerbated by inflammatory responses during infection. Proinflammatory cytokine release and EBOV-induced apoptosis can increase BBB permeability, allowing infected macrophages or free viral particles to infiltrate the brain parenchyma [13,14]. Recent work also suggests that treatment with monoclonal antibodies may promote infection of circulating monocytes that subsequently traffic into the brain, serving as a potential “Trojan horse” route of viral entry [15]. Findings from nonhuman primate survivors of EBOV exposure demonstrated BBB breakdown, macrophage infiltration, and viral persistence within the brain ventricular system, reinforcing the concept of direct CNS invasion [15]. Expression of EBOV VP40 in apoptotic neurons of the brainstem further supports potential neuronal involvement in pathogenesis [8].

Understanding how EBOV enters the CNS is crucial to preventing and treating the long-term neurological consequences faced by survivors. Detection of EBOV in cerebrospinal fluid (CSF) and brain tissue has been linked to seizures [16], cognitive dysfunction [6], and psychiatric disturbances [16], yet the precise mechanisms of viral entry remain unclear. Uncovering these pathways could reveal critical windows for therapeutic intervention, shape postinfection monitoring protocols, and ultimately reduce the burden of chronic neurological disease in EVD survivors.

## Q2: When does Ebola virus reach the brain, and under what conditions?

Understanding the timing and circumstances under which EBOV reaches the CNS is crucial for deciphering the pathogenesis of long-term neurological sequelae. Evidence from both clinical and experimental studies suggests that neuroinvasion can occur during both the acute phase of infection and in the post-acute setting, depending on host immune status, viral load, and integrity of the BBB [6,15,16].

In the acute phase, high systemic viral loads and a surge of proinflammatory cytokines are thought to promote BBB breakdown, enabling viral particles or infected immune cells to enter the CNS. In nonhuman primates, EBOV infection triggers profound inflammation and vascular leakage within the brain, consistent with BBB compromise [8]. In patients, viral RNA and antigens have primarily been detected in CSF, rather than in direct CNS tissue. While some patients exhibit signs of meningoencephalitis before complete resolution of systemic symptoms, EBOV can also access or persist in the CNS after the resolution of acute symptoms. Detection of viral RNA in CSF weeks to months after recovery supports the hypothesis that the virus either enters the brain late or persists within CNS-associated sites such as the choroid plexus, meninges, or resident cells, including astrocytes and neurons, or immune cells like microglia and macrophages [1,4,13]. Similar to other neurotropic RNA viruses, such as measles virus in subacute sclerosing panencephalitis, EBOV may persist within immune-privileged sites through low-level replication or direct cell-to-cell spread without abundant virion production, allowing evasion of immune clearance and long-term viral RNA detection [17]. Such persistence is often associated with delayed onset of neurological symptoms, including seizures, cognitive impairment, and fatigue [15].

Several factors may influence whether and when EBOV invades the CNS. These include individual variability in immune responses, viral strain differences, and co-morbid conditions that affect vascular permeability [4,13]. Emerging biomarkers, such as elevated viral loads in CSF or patterns of neuroinflammation, could eventually aid in predicting CNS involvement and long-term risk [1]. The temporal variability of EBOV neuroinvasion underscores the need for longitudinal monitoring of survivors, as neurological symptoms may arise long after systemic clearance of the virus.

## Q3: Which cells serve as Ebola viral reservoirs in the brain, and how might they contribute to persistence and relapse?

Once EBOV gains access to the CNS, identifying which cell types act as viral reservoirs becomes critical to understanding the mechanisms of neurological persistence and post-recovery relapses. Several cell types within the brain have been proposed as possible viral reservoirs. Among them, microglia have garnered the most attention, as they are the resident macrophage-like immune cells of the CNS and have been shown in some models to harbor viruses during infection. However, reports remain inconsistent across studies, and it is still unclear which brain-resident cells are truly susceptible to EBOV infection and capable of supporting long-term viral persistence. Beyond microglia, neurons and astrocytes [13] have also been proposed as potential targets based on limited experimental and histopathological data. The lack of consensus highlights the complexity of studying EBOV neuropathogenesis and underscores the need for more definitive evidence.

This gap is especially compelling in light of reports that viral RNA or antigens can linger in the CNS well beyond the resolution of acute symptoms. These findings raise important questions about whether EBOV establishes true latency in specific cell populations or instead persists at low replicating levels. Understanding which cells are permissive to infection, and whether they support active replication or serve as sanctuaries, is essential to understanding how the virus contributes to chronic neurological symptoms and relapse.

At present, there is no clear answer as to whether EBOV persistence in the brain is primarily due to direct infection of specific cell types, ongoing low-level replication, or immune-mediated disruption following initial infection. Clarifying the identity and function of potential reservoir cells will be key to developing therapeutic strategies aimed at eliminating persistent virus and mitigating long-term CNS damage. These questions are especially urgent given the unpredictability of EBOV outbreaks and the risk of future epidemics where postinfection sequelae may impose significant public health burdens.

## Q4: How should evidence of CNS involvement in EVD shape survivor care, diagnostics, and future research priorities?

Recognition of CNS involvement in EVD survivors has major implications for clinical care, diagnostic strategies, and research planning. As evidence accumulates for EBOV persistence in brain regions and its association with long-term neurological symptoms, a more proactive, multidisciplinary approach is needed to address survivor needs.

### Survivor care and clinical monitoring

Many EVD survivors experience cognitive dysfunction, seizures, depression, and chronic headaches well beyond viral clearance from peripheral blood [1,18]. As such, comprehensive neurological evaluations should be integrated into post-EVD care. These should include cognitive assessments, psychiatric screenings, and, where feasible, baseline and follow-up neuroimaging [4,19]. Survivors treated with immunotherapies, including convalescent plasma, may require additional surveillance due to potential immune-related sequelae [6].

### Diagnostic strategies

Standard diagnostic tools often fail to detect CNS involvement until symptoms are overt. Incorporating CSF analyses can reveal viral RNA or inflammatory markers that may otherwise go undetected [16]. Advanced imaging techniques such as MRI and PET, coupled with biomarker profiling of neuroinflammation, could improve early identification of EBOV-related neurological damage [8]. However, access to these tools remains limited in many outbreak regions, highlighting a key implementation gap and barrier to optimal diagnostic strategies.

### Research priorities

Future studies must clarify the mechanisms by which EBOV persists in the CNS and triggers chronic neuroinflammation. This includes investigating the role of glial cells, immune modulation, and potential other latent reservoirs. Longitudinal survivor studies are essential to define the full spectrum and duration of neurological sequelae [18,19]. Development of therapeutic strategies, such as antivirals with CNS penetration or anti-inflammatory agents targeting microglial activation, may help mitigate long-term cognitive and functional impairments [8].

### Ethical and logistical considerations

Research on CNS involvement in EVD also raises substantial ethical and logistical challenges. Performing lumbar punctures in resource-limited settings may be constrained by infrastructure, personnel, and cultural acceptability, as seen in both EVD and other neuroinfectious diseases such as HIV-associated cryptococcal meningitis [20]. Accessing brain tissue in survivors is ethically untenable, limiting mechanistic insights primarily to fatal case autopsies or animal models [21]. Even in fatal cases, full cranial autopsies are exceedingly rare, as skull opening poses a significant biosafety hazard where bone fragments can compromise PPE integrity, further restricting direct neuropathological investigation. Moreover, disparities in long-term neurological care, including access to mental health services, neuroimaging, and follow-up, are well-documented in Western African EVD cohorts [22]. Addressing these gaps will require collaborative, interdisciplinary strategies to ensure survivor-centered research and care.

## Q5: What are the strengths and limitations of animal models in elucidating EBOV’s neurological impact?

Current models of EVD have significantly advanced our understanding of systemic viral replication and immune dysregulation, but remain limited in their ability to capture the complexity of neurological involvement. Most studies report CNS viral RNA or antigen late in infection, yet few models offer high-resolution insight into how EBOV disrupts the brain across time, which cell types are affected, and how this correlates with long-term functional outcomes. Improving model systems is essential to better mimic human disease and uncover the mechanisms that drive neurological sequelae in EVD survivors.

Several methodological improvements could address these gaps:

- **Neuroimaging-based approaches:** Longitudinal MRI and PET imaging can noninvasively track BBB disruption, inflammation, and neural injury in vivo. These tools have been effective in SARS-COV-2 [23] and HIV [24] models to monitor neuropathology.
- **Behavioral testing batteries:** In mice, assays like the Morris water maze [25], open field test, elevated plus maze, and fear conditioning detect subtle deficits in cognition and behavior [26]. However, long-term behavioral assays, especially those requiring repeated handling or large apparatuses like the water maze, are generally impractical in BSL-4 settings due to biosafety restrictions, stress-related confounders, and logistics. Alternatively, cognitive performance in animals and survivors can be assessed with standardized neurocognitive tests, allowing cross-species comparisons and linking infection or stress to cognitive deficits.
- **Mapping sites of CNS entry:** The choroid plexus is a proposed entry site for EBOV. Future work could combine BBB permeability assays, viral exposure of choroid plexus epithelial cells, and tissue imaging to assess this pathway.
- **Tracking immune cell entry:** Monocytes and macrophages may carry virus into the brain. Imaging-based tracking across the choroid plexus could clarify their role in CNS viral invasion [14].
- **Defining infected brain cell types:** FISH, confocal microscopy, and flow cytometry can identify infected cells with spatial and temporal resolution. These tools could resolve which cell types (e.g., microglia, neurons, astrocytes) are involved over time.
- **Real-time infection tracking:** Luciferase-tagged recombinant vesicular stomatitis virus encoding the Ebola virus glycoprotein (rVSV-EBOV-GP) viruses allow *in vivo* imaging of infection spread. This approach improves temporal precision and reduces reliance on fixed endpoint analyses.
- **Mouse model manipulation:** Using sublethal EBOV doses (e.g., LD₅₀) allows long-term tracking of survivors beyond acute illness. This enables assessment of viral persistence, delayed pathology, and post-symptom neurological changes.
- **New Approaches Methodologies (NAMs) and in vitro platforms**: NAMs, including organotypic brain slice cultures, cerebral organoids, and BBB organ-chips, are increasingly applied to model EBOV neuroinvasion and host responses under controlled conditions. These systems provide high-resolution insight into virus–host interactions while mitigating the logistical and biosafety constraints of BSL-4 animal studies [27–29].

Integrating these animal model tools with longitudinal survivor cohorts in humans (e.g., PREVAIL III [22], PostEbogui [30]) will be critical for aligning mechanistic findings with human outcomes. Bridging model systems with clinical-neurological data will ultimately guide the development of diagnostics and therapies for EVD survivors facing CNS complications. While these approaches hold strong potential, their implementation under BSL-4 containment presents practical challenges: long-term survival and behavioral studies are logistically demanding, and sublethal infections may not fully recapitulate CNS involvement observed in severe disease. Nonetheless, adapting these strategies through pseudotyped viral systems for entry studies or recombinant surrogate models such as rVSV-EBOV-GP can help overcome feasibility barriers while maintaining biosafety compliance. Beyond clinical outcomes, understanding viral persistence in the CNS also carries major public health implications, as persistent infection and immune activation can contribute to stigma, hinder survivor reintegration, and, in rare cases, enable sexual transmission or outbreak reignition.

Addressing these facets is essential to holistic care, surveillance, and outbreak prevention strategies for EVD. Future investigations should aim to clarify the cellular and molecular mechanisms that enable EBOV persistence within the CNS, including whether viral latency or intercellular spread contributes to long-term infection and neurological sequelae.
